# Clinical utility of transperineal template-guided mapping prostate biopsy in a selection of patients under active surveillance and confirmation of patients with negative biopsy for prostate cancer

**DOI:** 10.3389/fonc.2024.1403237

**Published:** 2025-03-14

**Authors:** Michael Jakun Koo, Byunghun Lee, Wan Song, Minyong Kang, Hyun Hwan Sung, Byong Chang Jeong, Seong Il Seo, Seong Soo Jeon, Chung Un Lee, Hwang Gyun Jeon

**Affiliations:** ^1^ Department of Urology, Samsung Medical Center, Sungkyunkwan University School of Medicine, Seoul, Republic of Korea; ^2^ Department of Urology, Chung-Ang University Gwangmyeong Hospital, Chung-Ang University College of Medicine, Gwangmyeong, Gyeonggi-do, Republic of Korea

**Keywords:** prostate cancer, active surveillance, transperineal biopsy, multiparametric MRI, transrectal ultrasound guided biopsy

## Abstract

**Purpose:**

We investigated the change to definitive treatment in patients under active surveillance (AS) and cancer diagnosis in non-cancerous patients for prostate cancer after confirmatory transperineal template-guided mapping biopsy (TTMB).

**Materials and methods:**

A total of 336 patients who underwent TTMB between March 2017 and March 2023 were retrospectively reviewed, with 134 AS patients and 202 non-cancerous patients. All patients were routinely followed up via prostate-specific antigen (PSA) and multiparametric magnetic resonance imaging (mpMRI), and follow-up biopsy was performed when deemed clinically appropriate. Treatment changes in the AS and cancer detection in the non-cancerous group were analyzed. Descriptive statistics were used to analyze the retrospective data, and the Kaplan–Meier analysis was performed to indicate conversion to radical treatment in the AS group, as well as cancer detection in the previously benign non-cancerous group.

**Results:**

One hundred thirty-four patients under the AS protocol were analyzed, of whom 110 (82.1%) maintained AS for 33 months. Nine patients (6.7%) had significant findings in mpMRI [Prostate Imaging–Reporting and Data System (PI-RADS) ≥3] and received radical treatment following target biopsy via transrectal ultrasonography. A total of 115 patients (83.3%) with insignificant findings in mpMRI (PI-RADS 1 or 2 lesions) were followed up via transrectal ultrasound-guided prostate biopsy (17.4%, N = 20), repeat TTMB (6.1%, N = 7), or no additional biopsy (76.5%, N = 88), and from each group, five (25.0%), two (28.5%), and eight (9.1%) patients converted to radical treatment. In the non-cancerous group, five patients (2.5%) were diagnosed with prostate cancer, with PI-RADS ≥ 3 findings via mpMRI, and were confirmed by target biopsy during a mean follow-up period of 25 months, subsequently receiving radical treatment.

**Conclusions:**

TTMB is effective in selecting patients for AS treatment and confirming benign patients and can be used as an effective follow-up modality.

## Introduction

Prostate cancer (PCa) is currently one of the most diagnosed cancers worldwide and in South Korean men, particularly due to increased average life expectancy, dietary changes, and increased cancer screening (1, 2). It is expected to be one of the most diagnosed cancers in the near future (3). Various modalities are used for PCa screening, whereby the most prevalent and useful screening tool is prostate-specific antigen (PSA). Patients with elevated PSA undergo prostate biopsy, most commonly via transrectal ultrasound-guided prostate biopsy (TRUS-bx). However, there are several limitations of TRUS-bx: 30% of PCa is found at the anterior prostate and cannot be reliably targeted (4). Moreover, patients have a 2%–7% chance of post-biopsy sepsis (5), and repeat TRUS-bx has decreased cancer detection rates (6), affecting overall accuracy.

Transperineal template-guided mapping biopsy (TTMB) has been introduced to overcome these limitations by increasing the number of biopsy cores and targeting the anterior aspect of the prostate that would have been otherwise missed by TRUS-bx, significantly decreasing the possibility of post-biopsy sepsis by avoiding transrectal approach (5, 7–11). TTMB’s comprehensive method of biopsy allows for higher accuracy in obtaining biopsy cores from the prostate. TTMB has some limitations, mainly that it requires general anesthesia and may result in post-operative acute urinary retention (AUR), especially in patients with larger prostate volumes (12, 13). Recent studies on the feasibility of performing TTMB under local anesthesia have reported positive results that can be incorporated into local clinical settings without compromising accuracy (14, 15), although the subjectivity of post-procedural pain and its severity is non-uniform, and thus, patients may prefer the procedure under general anesthesia, mostly those who require increased biopsy cores due to larger prostate size (16).

Various modalities are available for PCa diagnosis and treatment. Particularly, early-stage localized PCa has an indolent nature that allows for non-radical treatment without impacting overall survival (17). According to the American Urological Association/American Society for Radiation Oncology (AUA/ASTRO) guidelines, active surveillance (AS) can be considered as a treatment option for patients grouped into low and favorable intermediate risks, where the low-risk group is identified as PSA < 10 ng/mL, Gleason score grade group 1, and cT1–cT2a (18).

As stated by the guideline, biopsy results are one of the significant criteria for selecting patients for AS. Additionally, coupled with the advantages of TTMB, such as a wide-ranging approach and attainment in number and location biopsy cores (19–21) as well as decreased risk of sepsis, TTMB can be utilized as an effective modality for selecting and monitoring patients with prostate who are eligible for AS. Additionally, TTMB can act as a confirmatory modality in previous biopsy-naïve patients for screening and monitoring. Comprehensively, TTMB can be used as an effective tool in screening for clinically significant prostate cancer (csPCa), determining eligibility for AS protocol, and monitoring patients for disease development and/or progression.

There are limited reports and guidelines, however, that emphasize the role of TTMB in follow-up protocol for AS patients and confirm the absence of cancer in patients who are strongly suspected of PCa. We aimed to illustrate the clinical utility of TTMB in selecting patients for AS and its usefulness in detecting non-cancerous lesions in previously negative TRUS-bx patients in a clinical follow-up setting.

## Materials and methods

### Aim

This study aimed to, first, identify the clinical course of patients of clinically insignificant PCa who were treated via AS following confirmatory TTMB and, second, display the clinical follow-up course of non-cancerous patients following confirmatory TTMB.

### Design and population

This study was approved by the Institutional Review Board of Samsung Medical Center (2024-02-119-001) and was performed in accordance with the principles of the Declaration of Helsinki. We retrospectively reviewed a database of 352 patients who underwent TTMB between March 2017 and March 2023 at the Samsung Medical Center. We divided the patients into two groups: the AS (N = 150) group and the non-cancerous group (N = 202). We excluded a total of 16 patients who were lost to follow-up. We used low-risk (PSA < 10 ng/mL and grade group 1 and cT1a–cT2a) and favorable intermediate-risk (grade group 1 with PSA of 10–20 ng/mL or cT2b–cT2c and <50% biopsy cores positive or grade group 2 with PSA < 10 ng/mL and cT1–cT2a with <50% biopsy core positive) patients, as outlined by the AUA/ASTRO guidelines, to determine the eligibility of the patient for AS treatment. All patients received confirmatory TTMB, whether for confirming the validity of AS treatment or the presence of PCa in patients with elevated PSA levels. We performed TTMB should the patients be eligible for certain criteria, such as persistently high PSA levels or a trend of increasing PSA levels despite negative biopsy results on previous biopsies, or patients were diagnosed with low-risk PCa and were, therefore, suitable candidates for AS. We also performed TTMB should patients specifically request this biopsy method. We routinely followed up all patients via PSA levels and multiparametric MRI (mpMRI) should it be deemed necessary by the clinician. Patients received repeat target-bx by TRUS should follow-up mpMRI reveal Prostate Imaging–Reporting and Data System (PI-RADS) lesion of ≥3, while we followed up patients with insignificant mpMRI results (PI-RADS 1 or 2 lesions) clinically or received repeat biopsies for various reasons, such as patient preference, persistent PSA level, and newly elevated PSA level.

### Data collection and analysis

We collected clinical, pathological, imaging, and laboratory data of all our patients, including age, initial PSA level, prostate volume, Gleason score, number of positive and total cores, and mpMRI findings reviewed and dictated by a radiologist with a specialty in the genitourinary field. All biopsy specimens were reviewed by a pathologist with a specialty in genitourinary oncology.

### TTMB technique

All TTMB procedures were performed under general anesthesia, and biopsy was conducted by a urologist. Patients were placed in the lithotomy position, and a transrectal ultrasound probe was inserted to measure the size of the prostate and confirm the presence of hypoechogenic lesions. Prostate MRI findings, should they be available, were consulted during this procedure. Patients underwent either 24- or 36-core systemic biopsy, depending on the size of the prostate, with a normal range of ≤30 cc, via the Ginsburg protocol (22). Should the MRI findings and/or hypoechogenic lesions be visible, two or more target biopsies were performed at the operator’s discretion.

### Follow-up protocol

We recommended that patients undergo an MRI once every 1 to 2 years and prostate biopsy every 2 to 5 years, with intervals between biopsies being no less than 12 months as the guidelines suggested (23, 24). We also did not recommend follow-up biopsies within a 12-month span, and we recommended follow-up biopsy time period depending on each patient’s tumor grade, tumor volume, and/or changes in PSA trend. Some patients rejected follow-up biopsies despite our recommendations, for which we followed up the patients via mpMRI, and we explained that future biopsies may be required depending on the results of follow-up modalities. In the non-cancerous group, we performed follow-up biopsies in patients with a trend of maintaining or increasing PSA levels over 1 year.

### Statistical analysis

Quantitative variables were presented as medians [interquartile range (IQR)] or means (standard deviation), and qualitative variables were presented as absolute values (%). Descriptive statistics were obtained for demographic variables. The Kaplan–Meier analysis was performed to indicate the radical treatment rate in the AS group as well as the cancer-free rate in the non-cancerous group during the follow-up period. All statistical analyses were performed using IBM SPSS for Windows, version 23.0 (IBM Corp., Armonk, NY, USA).

## Result

**Figure 1:**
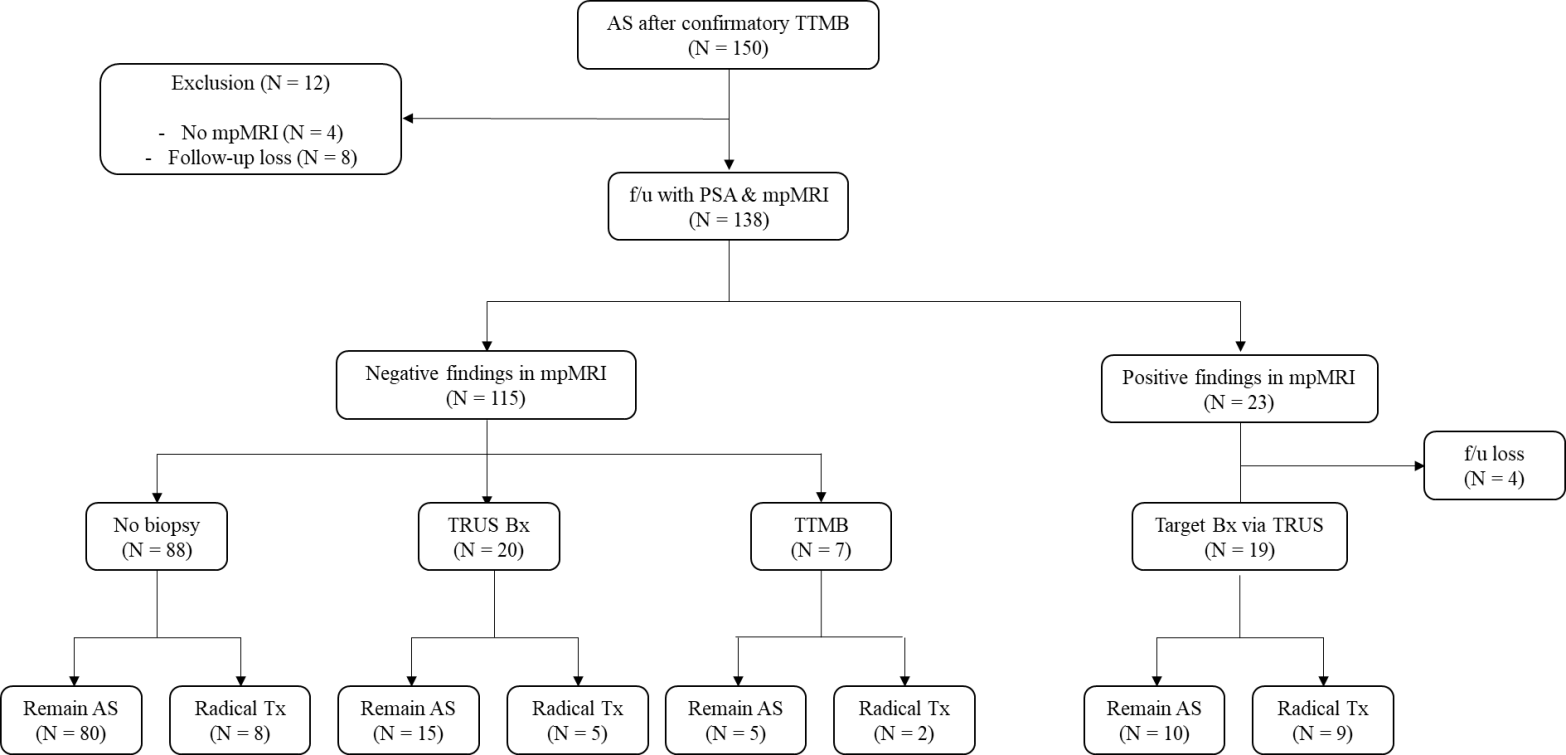
Flowchart of the AS group. Of the 150 clinically insignificant patients with PCa, 138 underwent AS, while 12 patients dropped out before further screening tests. The remaining patients were further divided into two groups: those with significant findings in mpMRI (N = 23) and those without significant lesions in mpMRI (N = 115). Those without significant lesion findings in mpMRI were divided into those who received follow-up biopsy (TRUS-bx, N = 20, and TTMB, N = 7) and those who did not receive biopsy (N = 88). Patients who received radical treatment and those who continued AS are as follows. Patients with mpMRI(+) (N = 19) received target TRUS-bx, of whom nine patients received radical treatment. mpMRI, multiparametric MRI; AS, active surveillance; TTMB, transperineal template-guided mapping biopsy; f/u, follow-up; Bx, biopsy; Tx, treatment; PI-RADS, Prostate Imaging–Reporting and Data System. Positive mpMRI findings are PI-RADS lesions of 3 or higher.

Baseline patient characteristics are shown in **Table 1**. The average age of patients in the AS and non-cancerous groups was 66 and 62 years, respectively. The mean PSA of each group was 5.79 and 8.97, with a median (IQR) of 4.90 (3.5–7.1) and 6.62 (4.7–10.4). The mean follow-up period of each group was 33.8 and 32.9 months. The total number of patients who discontinued AS was 28 (20.3%), with an average intervention time of 24 months. In the non-cancerous group, the number of patients who received re-biopsy was nine (4.5%), of whom five patients (2.5%) ultimately received radical treatment.A total of 150 patients in the AS group initially participated in this study. After excluding 12 patients, eight patients due to being lost to follow-up and four patients due to mpMRI was not performed, the remaining 138 patients were further divided based on findings of follow-up mpMRI: 115 (83.3%) patients had insignificant lesions (PI-RADS lesion 1 or 2), whereas 23 patients (16.6%) had significant lesions (PI-RADS ≥ 3). Of the 23 patients, four (17.4%) were lost to follow-up, and the remaining 19 (82.6%) underwent target biopsy via TRUS, of whom nine (47.4%) ultimately received radical treatment (due to disease progression). Patients without significant findings in mpMRI were further divided into three groups based on clinical treatment course: those followed up without biopsy (76.5%, N = 88) and those followed up via biopsy, via either TRUS-bx (17.4%, N = 20) or TTMB (6.1%, N = 7). The number of patients who received radical treatment from each group was eight (9.1%), five (25%), and two (28.6%). Of 138 patients, 24 (17.4%) ultimately underwent radical treatment (16 radical prostatectomy, 6 radiation, 1 partial gland ablation, and 1 radical cystectomy due to concurrent diagnosis of bladder cancer), with a mean time to intervention of 24.0 months (**Figure 1**).

**Table 1:** Baseline characteristics.

Variable	AS group	Non- cancerous group
No. of patients, n (%)	134	202
Age at biopsy (years), median (IQR)	66 (61–70)	62 (56–66)
PSA (ng/mL), median (IQR)	4.90 (3.5–7.1)	6.62 (4.7–10.4)
Prostate volume (cm^3^), median (IQR)	38.5 (30–50.1)	44.1 (33.2–56.6)
No. of positive cores in TRUS-guided biopsy		
Median (range)	1 (1–3)	–
Gleason grade in TRUS-guided biopsy, n (%)		
6	112 (74.7%)	–
7 (3 + 4)	4 (2.6%)	–
Follow-up period (months), mean	33.8	32.9
Discontinuation of AS, patient no., mean	28 (20.3%)	–
Time to intervention, months, mean	24.0 (5.6–48)	–
No. of patients receiving re-biopsy (%)	–	9 (4.5%)
Prostate cancer diagnosis rate, patient no., (%)	–	5 (2.5%)

TRUS, transrectal ultrasound; AS, active surveillance; IQR, interquartile range; SD, standard deviation.

**Figure 2:**
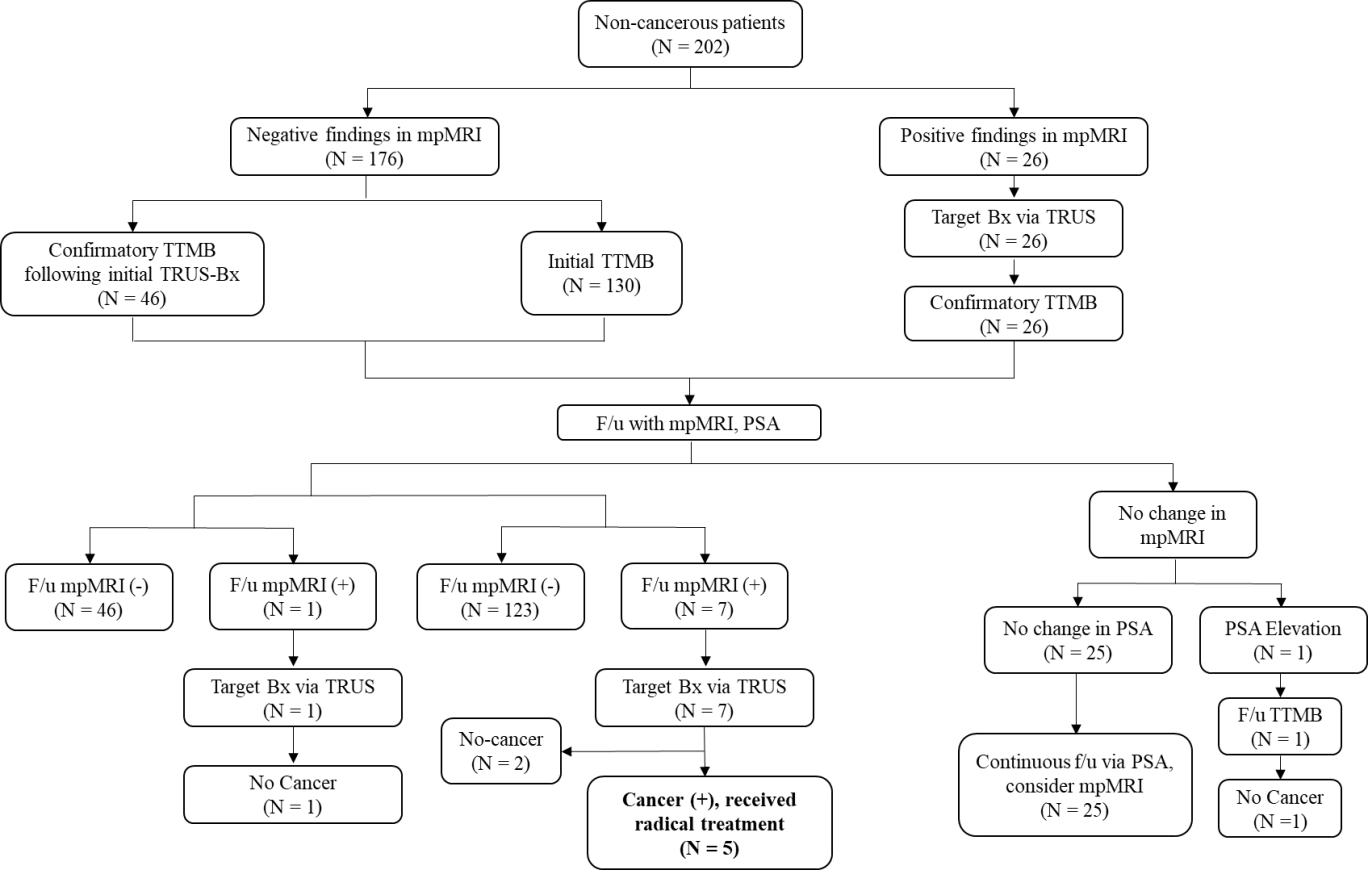
Flowchart of the non-cancerous patients. All patients underwent mpMRI, and those with significant mpMRI findings (N = 26) underwent target Bx via TRUS, subsequently undergoing confirmatory TTMB. Patients who received initial TRUS-bx (N = 46) also underwent subsequent confirmatory TTMB. All patients who did not have cancer were then followed up via PSA and mpMRI, after which a similar f/u protocol was followed based on mpMRI findings. Eight patients with significant mpMRI findings underwent target bx via TRUS, five of whom were then newly diagnosed with csPCa, for which they received radical treatment. TTMB, transperineal template-guided mapping biopsy; f/u, follow-up; bx, biopsy; Tx, treatment; csPCa, clinically significant prostate cancer; PSA, prostate-specific antigen.

A total of 202 patients from the non-cancerous group received initial TTMB (64.3%, N = 130) or confirmatory TTMB after the initial TRUS biopsy (35.6%, N = 72). Subsequently, these patients were followed up via PSA levels and mpMRI and were further divided based on those who did not receive mpMRI (83.2%, N = 168), those with significant findings in mpMRI (3.9%, N = 8), and those with insignificant findings in follow-up mpMRI (12.8%, N = 26). One patient (3.8%) from the group with insignificant findings received a repeat follow-up biopsy due to PSA elevation via TTMB and was ultimately not diagnosed with PCa. Eight patients (3.9%) with significant mpMRI findings underwent target bx via TRUS, of whom five (62.5%) were newly diagnosed with csPCa, leading to radical treatment (**Figure 2**).

**Figure 3:**
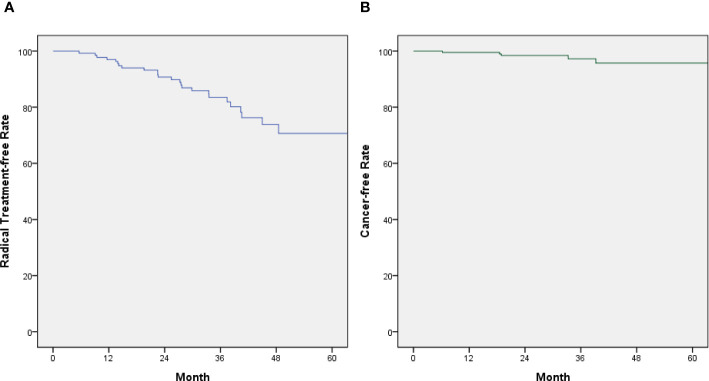
**(A)** Kaplan–Meier graph depicting the patients from AS group who received radical treatment (blue). Radical treatment-free rates of the AS group for the first, second, and third years were 96.3%, 91.0%, and 85.8%, respectively. **(B)** Kaplan–Meier graph depicting the patients of the non-cancerous group who presented cancer-free rates (green). Cancer-free rates of the non-cancerous group for the first, second, and third years were 99.5%, 98.5%, and 98.0%, respectively.

The patients who ultimately received radical treatment from the AS and non-cancerous groups are illustrated via the Kaplan–Meier curve (**Figure 3**), which shows the radical treatment-free survival curve in the AS and non-cancerous groups. Radical treatment-free rates of the AS group for the first, second, and third years were 96.3%, 91.0%, and 85.8%, respectively (**Figure 3A**), while the cancer-free rates of the non-cancerous group for the first, second, and third years were 99.5%, 98.5%, and 98.0%, respectively (**Figure 3B**).

## Discussion

Traditionally, the mainstay protocol of the biopsy method for PCa screening is conducted via 12-core TRUS biopsy. Several studies have shown the significance and suitable application of mpMRI in aiding PCa detection and diagnosis (25, 26). Therefore, many more studies have illustrated the advantages of MRI-targeted biopsy (27–29), leading to its place as one of the default screening methods for PCa. Despite remarkable advancements in PCa diagnostic tools and modalities, studies have shown that a fraction of patients with PCa were not diagnosed and/or did not receive proper treatment based on the staging of PCa at diagnosis (4, 5). Studies regarding TTMB have demonstrated its effectiveness in detecting significant PCa, and many studies from our institution have illustrated the effectiveness of TTMB compared to other biopsy methods (19–21). Thus, we aimed to demonstrate the role of TTMB and its efficacy during the clinical course of patients under AS and non-cancer patients.

A study by Song et al. showed long-term follow-up data of AS patients for 15 years, in which 38% of patients underwent radical treatment (30), while Ha et al. reported 48.6% of AS patients received radical treatment over a follow-up of 4 years (31). Patients in both of the aforementioned studies underwent TRUS-bx and were subsequently treated via AS based on pathology obtained from the TRUS-bx cores. Compared to these studies, our study showed that 17.4% of patients underwent radical treatment over a mean follow-up period of 33.8 months. Our study design incorporated more strict criteria in determining AS eligibility by performing confirmatory TTMB after initial TRUS-bx or target bx via TRUS, thus having an additional layer of the screening process, therefore resulting in a markedly low percentage of patients converting to radical treatment. Patients who received confirmatory TTMB did not present inferior results, regarding the number of patients converting to radical treatment, compared to those who received only TRUS-bx.

As for whether repeat biopsies reveal PCa that was previously undiagnosed in initial biopsies, Ploussard et al. showed that initially, repeat biopsies of non-cancerous patients during a mean time follow-up of 19 months revealed 16.7% of newly diagnosed patients with cancer, with similar results for repeat biopsies (32). Our study showed that a total of five patients (2.5%) converted to radical treatment over a mean follow-up period of 31 months, with one repeat biopsy performed. A further study by Patel et al. showed that patients with previous negative biopsies along with utilization of mpMRI had 5.3% detection of csPCa in a 5-year follow-up span (33). Our findings, along with those of aforementioned studies, show the benefits of TTMB and mpMRI in that by implementing and adhering to stricter criteria, patients can be screened with higher accuracy, leading to scheduling patients for a much less intense follow-up course, with fewer clinical visits and invasive tests performed than otherwise indicated or traditionally applied.

TTMB has many advantages over systematic TRUS-bx: confirming the eligibility of AS for PCa patients and holding a firm rationale for undergoing confirmatory biopsy in patients suspected of PCa. TTMB’s higher number of biopsy cores collected allows for a broader area of the prostate that could be examined, which can detect cancer in regions that would have been otherwise missed by a systematic 12-core biopsy. Additionally, the risk of post-biopsy urinary tract infection and urosepsis is significantly lower, which can lessen the medical cost and health burden for the patients. Moreover, performing a more extensive biopsy method such as TTMB allows for a more comprehensive and accurate diagnosis of the patient’s status at the time of the clinic visit, which allows for a more lenient follow-up term to monitor the patient’s condition.

However, TTMB is often performed under general anesthesia, and care must be taken to maintain sterility and the accuracy of such invasive tests. Although the risk of post-biopsy complications and infection is lower in TTMB compared to that of the transrectal approach, there exists the possibility of post-biopsy AUR, especially in patients with larger prostate volumes (12, 13, 19), underscoring the need for careful selection of biopsy modality for patients regarding prostate volumes. Additionally, an extended follow-up duration can be considered for patients who underwent confirmatory TTMB, as opposed to patients who received traditional TRUS-bx. Although our report does not specifically demonstrate the clinical course of increasing follow-up duration, incorporation of such measures into the clinical protocol, based on the rate of conversion to radical treatment from AS and detection of csPCa of non-cancerous patients, does not seem far-fetched. Moreover, extending follow-up duration can be favorable for the patients, as this would lead to less invasive tests performed on patients, improving the patient’s overall quality of life and sparing additional medical costs.

Recent studies have highlighted the feasibility of performing TTMB under a local anesthetic setting, with results that are comparable to those of TTMB conducted under a traditional, general anesthetic setting (34), which aids in maintaining the same level of excellent accuracy while lessening the burden on the patients. Recent studies have also proposed various methods of applying local anesthesia to provide the least discomfort and pain in exchange for eliminating general anesthesia, with promising results (35, 36). Our study could not incorporate such tactics, but transperineal biopsy under local anesthesia can be the basis of future studies when fully integrated at our institution. Overall, the advantages of transperineal biopsy can minimize the rate of conversion to radical treatment in AS patients and csPCa detection in previously benign, non-cancerous patients.

Additionally, as for the ideal approach of TTMB, we believe that performing target biopsy along with TTMB should be the best option in selecting a stricter standard for AS treatment in csPCa patients. Because biopsy results from TTMB can either yield cancerous cells in regions that were found to be benign in mpMRI or yield cancerous cells with upgraded GS (37, 38), performing TTMB with target biopsy can optimize the biopsy method as well as the results, benefitting both the patient and the clinician with accurate and updated information that reflects patient’s condition (39). Previous studies that compared the detection rates of csPCA with TTMB versus mpMRI/ultrasound fusion target biopsy have demonstrated that target biopsy alone has a lower detection rate of csPCa than TTMB (40, 41). Another study has shown that target biopsy using a transperineal approach allowed for higher detection of csPCA than the transrectal approach, further highlighting the advantages of TTMB as well as target biopsy (42).

Our study had some limitations. First, the retrospective nature of this study means that selection bias that could affect our results may have occurred. Second, a relatively short follow-up period could have limited the overall scope of our study, as a longer follow-up period could have yielded different results. Additionally, our study was conducted at a tertiary teaching hospital in the most populous city in South Korea, which could cause the issue of generalizability to the general population of South Korea. Furthermore, all AS patients were seen by several different oncologists and therefore did not follow the same follow-up strategies regarding biopsy modalities and/or tests performed, and many factors, such as patient and doctor preferences, may have contributed to determining the clinical course regarding biopsy protocol (TRUS vs. TTMB). Overall, our study suggests the effectiveness of TTMB and its useful role in PCa screening as one of the screening and biopsy modalities.

## Conclusion

TTMB is clinically effective in confirmatory screening tests for AS of PCa and determining whether patients with clinically suspicious PCa are actual patients with cancer at risk for radical treatment. This study could assist in further establishing or creating a new set of guidelines that can consider using TTMB as a first-line biopsy modality for PCa screening.

## Data Availability

The original contributions presented in the study are included in the article/supplementary material. Further inquiries can be directed to the corresponding authors.
